# Bioactive flavonoids metabolites in citrus species: their potential health benefits and medical potentials

**DOI:** 10.3389/fphar.2025.1552171

**Published:** 2025-03-03

**Authors:** Yuqian Xu, Pan He, Beihui He, Zheng Chen

**Affiliations:** ^1^ The First Affiliated Hospital of Zhejiang Chinese Medical University (Zhejiang Provincial Hospital of Chinese Medicine), Hangzhou, China; ^2^ Sichuan Provincial Women’s and Children’s Hospital, The Affiliated Women’s and Children’s Hospital of Chengdu Medical College, Chengdu, China; ^3^ School of Life Sciences, Zhejiang Chinese Medical University, Hangzhou, China

**Keywords:** citrus, flavonoids, bioactivities, mechanisms of action, extraction techniques, health benefits

## Abstract

Citrus flavonoids are naturally occurring phytochemicals widely present in the peels and pulps of citrus fruits. They exhibit a wide range of biological activities, including antioxidant, anti-inflammatory, hypoglycemic, lipid-lowering, antimicrobial, and gut-protective effects. These metabolites show great potential in improving metabolic syndromes such as diabetes, non-alcoholic fatty liver disease (NAFLD), and cardiovascular diseases. Additionally, citrus flavonoids have demonstrated significant effects in inhibiting pancreatic lipase activity, regulating lipid metabolism, and enhancing intestinal barrier function. Advances in extraction and purification techniques have further promoted their applications in the fields of food, medicine, and functional materials. This review systematically summarizes the types, bioactivities, and mechanisms of action of citrus flavonoids, providing scientific evidence for their research and development.

## 1 Introduction

Citrus fruits, belonging to the subfamily *Aurantioideae* of the *Rutaceae* family, include oranges, grapefruits, lemons, limes, mandarins, and tangerines. They are considered one of the largest groups of plant species and are widely distributed across tropical, subtropical, and temperate regions of the world (Sharma et al., 2017). Research has confirmed that south-central China is a major origin center for the genus Citrus (Huang et al., 2023). Citrus fruits are rich in phytochemicals, particularly flavonoids, which represent an essential group of dietary flavonoids. The primary methods for extracting citrus flavonoids include solvent extraction and subcritical water extraction technology. These methods efficiently yield high-purity flavonoid metabolites from citrus, providing a solid foundation for subsequent research.

A substantial body of research indicates that citrus flavonoids possess a variety of biological activities, including antioxidant, anti-inflammatory, antibacterial, lipid-lowering, blood sugar-lowering, and intestinal protective effects. These natural plant chemicals show particular promise in improving metabolic syndromes such as diabetes, non-alcoholic fatty liver disease (NAFLD), and cardiovascular diseases. Moreover, the application of citrus flavonoids in the food industry is expanding, with the development of novel food packaging materials and their use as antioxidants in meat products, enhancing food safety and quality. In the field of pharmacology, citrus flavonoids like naringin and hesperidin demonstrate potential in treating various diseases, including overcoming drug resistance in cancer therapy. Therefore, in-depth research on citrus flavonoids is expected to provide more scientific evidence and therapeutic options for humanity.

## 2 Extraction methods of citrus flavonoids

### 2.1 Organic solvent extraction method

The organic solvent extraction method is based on the physical properties of flavonoids, selecting appropriate solvents to isolate different types of flavonoid metabolites. Commonly used solvents include methanol, ethanol, acetone, and ethyl acetate, with methanol and ethanol being the most commonly used. Methanol generally provides better extraction efficiency, followed by ethanol, but both solvents have higher extraction efficiency compared to other organic solvents. However, due to methanol’s toxicity and ethanol’s more environmentally friendly nature, ethanol is more commonly used. Phucharoenrak et al. (2022) used discarded sour orange peels from an acid orange juice factory as raw material, employing an ethanol-water extraction technique to obtain hesperidin and limonene. The study found that the optimal conditions for the highest yield were extracting with 80% ethanol at a solid-to-solvent ratio of 0.01 g/mL, pH 7, and 50°C for 100 min. Jeong et al. (2023) selected citrus junosas the raw material and employed solvent extraction combined with enzyme treatment techniques. The optimal conditions identified were 5% pomelo powder, 50% ethanol as the solvent, and 0.05% rhamnosidase treatment for 24 h. The mixture was purified using an HP-20 chromatography column, with 50% ethanol as the elution solvent, resulting in the highest concentrations of naringin and hesperidin. The organic solvent extraction method is cost-effective, has low toxicity, good reproducibility, and is suitable for industrial production (Bajkacz and Adamek, 2018).

### 2.2 Subcritical water extraction

Subcritical water extraction (SWE) is a novel extraction technology developed after supercritical fluid extraction. It uses subcritical fluids as the extraction solvent. Under certain pressures, extraction is carried out at temperatures ranging from 100°C to 374°C. This method offers several advantages, including easy solvent recovery with no residue, prevention of oxidation of the extracted products, preservation of the bioactivity of the extract, and low cost (Cheng et al., 2021). Kim and Lim (2020) employed a semi-continuous subcritical water extraction technique to extract flavonoids from citrus peel. Their study showed that under conditions of 145.3°C–165.6°C and a water flow rate of 2.25 mL/min, using water as the solvent, semi-continuous extraction yielded 87.8%–98.9% of bioactive flavonoids from the peel. Hwang et al. (2021) used discarded citrus peels as raw material to investigate whether combining subcritical water extraction with pulsed electric field (PEF) treatment could enhance the extraction efficiency of flavonoids from citrus. The results indicated that the extraction temperature and time significantly affected flavonoid extraction, with higher temperatures improving efficiency. The PEF treatment time also had a significant impact on the extraction of hesperidin. Compared to traditional extraction methods, subcritical water extraction is an efficient technique for extracting bioactive metabolites.

## 3 Types of flavonoid metabolites in citrus

To date, over 250 citrus flavonoid metabolites have been identified (Wang et al., 2019). Flavonoids are natural phenolic metabolites composed of two benzene rings and 15 carbon atoms. They can be broadly classified into six categories: flavanones, flavanols, flavones, flavonols, isoflavones, and anthocyanins (Peterson and Dwyer, 1998). Flavanones are the most abundant flavonoid metabolites found in citrus fruits, primarily existing in the forms of rutin glycoside and neo-hesperidoside. The flavonoids formed by the former are tasteless, such as naringin, hesperidin, pomelosidin, and melittoside, while those formed by the latter have a distinctly bitter taste, such as neo-naringin, neohesperidin, neo-poncirin, and rutinoside (Tripoli et al., 2007). Naringin, primarily found in grapefruit, may account for 30%–40% of the citrus flavonoids, while hesperidin, mainly present in the peels of oranges and other citrus fruits, typically makes up 20%–30%. In total, approximately 4,000 flavonoid metabolites have been isolated, with most of them found in fruits and vegetables. The concentration and distribution of citrus flavonoids vary significantly between species (Benavente-García and Castillo, 2008).

## 4 Health benefits of citrus flavonoids

**FIGURE 1:**
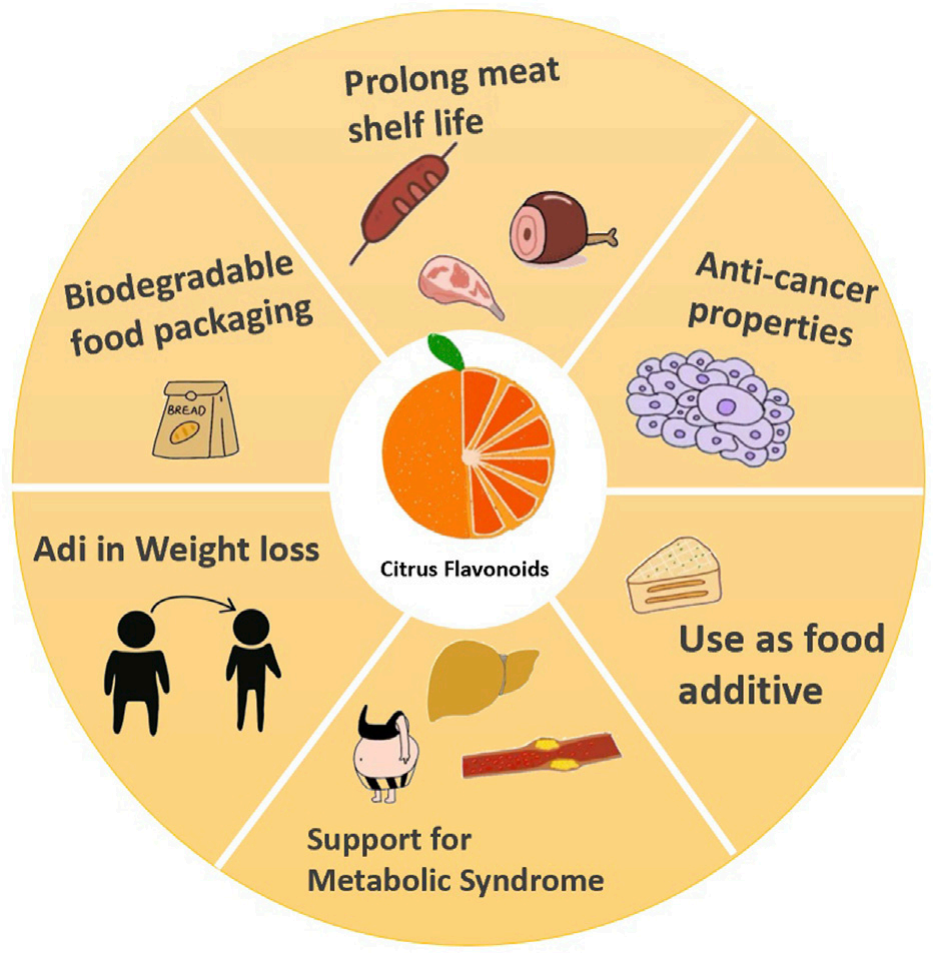
Health benefits of citrus flavonoids.

Citrus flavonoids are widely found in citrus fruits and offer significant health benefits. In the food industry, they serve as natural antioxidants to extend shelf life, while in pharmacology, they exhibit anti-inflammatory, anti-diabetic, and anti-cancer effects, playing a crucial role in human health (Figure 1).

### 4.1 Applications in the food industry

Food safety is the foundation of human health and is crucial to everyone’s daily life. With the development of technology, consumers’ demand for high-quality food has increased, making food packaging and quality hot topics.

There is widespread controversy and concern about the chemicals added to traditional plastics. Naringin is a flavonoid metabolite naturally found in citrus fruits, and it is widely distributed in the peels of fruits like grapefruit and oranges. Guzman-Puyol et al. (2022) developed a novel, transparent, UV-resistant, high-barrier, and biodegradable food packaging material made from a mixture of cellulose andnaringin. These bioplastics were prepared by dissolving the metabolites in trifluoroacetic acid (TFA) and trifluoroacetic anhydride (TFAA), followed by solvent removal via casting. Experiments showed that when 20% hesperidin was added (CN-20), the transparency remained over 85%, while the UV-B barrier reached 100%. In terms of mechanical properties, CN-20 exhibited a 25% increase in elongation at break, a 27% increase in toughness, and a reduction in Young’s modulus to approximately 1,500 MPa, demonstrating a significant plasticizing effect. The water vapor transmission rate (WVTR) decreased to 3,000 g/m^2^·day, and the oxygen transmission rate (OTR) dropped to 55 mL/m^2^·day, which is comparable to petroleum-based plastics such as polyester. Biodegradation testing in seawater showed that CN-20 had a biochemical oxygen demand (BOD) of 10 mgO2/L and a weight loss of 19%, indicating its suitability as a biodegradable packaging material in marine environments.

Free radical oxidation is one of the main mechanisms leading to the deterioration of food, especially meat products. Goliomytis et al. (2015) studied the addition of hesperidin or naringin to chicken feed, significantly improving the oxidative stability of breast and thigh meat. The study showed a dose-dependent decrease in the oxidative marker MDA levels, with the antioxidant effect of naringin approaching that of vitamin E, significantly extending the shelf life of the meat. These natural bioflavonoids may improve the antioxidant properties of meat by inhibiting lipid oxidation, providing a sustainable additive option for both consumers and the poultry industry. However, the extraction and purification costs of hesperidin and naringin are relatively high compared to common additives, which may impact their application in large-scale production.

### 4.2 Applications in pharmacology

Diosmin is a flavonoid glycoside derived from hesperidin. Huwait and Mobashir (2022) demonstrated that diosmin reduces oxidative stress by altering the activity of specific enzymes and promotes apoptosis in various cancer cell lines through multiple mechanisms. Its anti-inflammatory properties are attributed to its ability to lower the levels of several inflammatory markers. Diosmin also helps alleviate complications of diabetes, such as neuropathy and dyslipidemia. When combined with other flavonoids, especially hesperidin, diosmin is highly effective in treating chronic venous insufficiency and hemorrhoids. However, diosmin is a central nervous system depressant, and long-term use may trigger excitatory reactions, such as irritability, restlessness, dizziness, and headaches. Naringin has the ability to promote the conversion of white fat into beige fat, thereby enhancing the thermogenic capacity of fat and glucose, which aids in weight and blood glucose control. Studies have found that naringin activates genes that improve blood glucose control and increase calorie burning, making it a promising strategy for treating obesity and diabetes. Beige fat has thermogenic functions, accelerating metabolism, breaking down white fat, reducing fat storage, and improving glucose and cholesterol metabolism (Chen, 2025). Naringin is also an effective inhibitor of breast cancer resistance protein (BCRP), helping to overcome BCRP-mediated multidrug resistance, and is used as a P-glycoprotein (P-gp) inhibitor. The combination of naringin with paclitaxel enhances anticancer activity. The results showed better cytotoxicity against breast cancer cell lines, offering an effective approach to combating drug resistance (Jabri et al., 2019). Naringin also inhibits the expression of STAT-3, AKT, and Bcl-2, playing a role in resistance to chemotherapy. Treatment with naringin reduces tumor size in mice bearing tumors.

## 5 Medical potential of citrus flavonoids

Citrus flavonoids possess various biological activities, primarily including antioxidant, anti-inflammatory, antimicrobial, antidiabetic, lipid-lowering, cardiovascular protective, and gut protective effects (Table 1).

**TABLE 1:** Medical potential of citrus flavonoids.

Author (Reference)	Application fields	Major active metabolites	Mechanism of action	Specific effects
Ji (2019) Xiao et al. (2013)	Improvement of diabetes	Citrus flavonoids	Enhance antioxidant capacity, eliminate free radicals, improve lipid metabolism disorders, and regulate the expression of glucose metabolism genes through the Wnt signaling pathway	Reduce fasting blood glucose, improve insulin resistance, and regulate lipid levels
Guan et al. (2021) Zhang et al. (2021) Wu et al. (2017)	Improvement of NAFLD	Naringin	Activate autophagy pathways, promote lipid degradation, and reduce hepatic lipid accumulation	Significantly reduce TG levels, inhibit *de novo* lipogenesis, and improve liver function
Nakajima and Ohizumi (2019) He et al. (2021) Kim et al. (2016)	Neuroprotective	Citrus flavonoids	Inhibit β-secretase activity, promote amyloid protein degradation, activate the cAMP/PKA signaling pathway, enhance synaptic function, and reduce oxidative stress to alleviate neuroinflammation	Reduce amyloid plaque accumulation in Alzheimer’s disease (AD) and improve motor dysfunction and dopamine signaling in Parkinson’s disease (PD)
Heidary Moghaddam et al. (2020) Guo et al. (2022)	Cardiovascular protective	Naringin、Nobiletin	Inhibit oxidative stress, reduce cardiomyocyte apoptosis, activate the AMPK-mTOR pathway, decrease myocardial hypertrophy, and improve lipid profiles	Reduce atherosclerotic plaque formation, decrease myocardial damage, and inhibit the expression of inflammatory factors
Ferlazzo et al. (2016)	Antioxidant	Nobiletin、Naringenin	Increase the expression of antioxidant enzymes (SOD) and inhibit free radical generation	Reduce oxidative stress, protect cell membranes and mitochondria from damage
Chen et al. (2022) He et al. (2018)	Anti-inflammatory	Naringin、Naringenin	Inhibit the NF-κBpathway,reduce the expression of pro-inflammatory factors,and suppress cox-2andiNOS activity	Alleviate inflammatory damage and improve the tissue microenvironment
Dey et al. (2020)	Antimicrobial	Naringin	Enhance the efficacy of antibiotics (e.g., ciprofloxacin) and inhibit bacterial biofilm formation	Significantly clear *Pseudomonas aeruginosa* biofilm, reduce its extracellular matrix thickness, and protect healthy cells

### 5.1 Improvement of diabetes

Ji (2019) used the HepG2 cell-glucose consumption model to demonstrate significant differences in the hypoglycemic activity of different parts of citrus fruits. The active metabolites were primarily enriched in the oil vesicle and white peel layers of the fruit skin. Potential high-efficiency hypoglycemic citrus varieties such as “Ougan,” “Ehime 30,” “Chazhi Gan,” and “Amakusa” were identified, with the fruit peel oil vesicle layers being rich in polymethoxyflavones (PMFs). PMFs isolated from the peel of “Ougan” citrus significantly promoted glucose consumption and regulated the expression of glucose metabolism genes through the Wingless-integrated, inhibiting certain gene expressions and upregulating key regulatory factors. The physiological benefits of PMFs from “Ougan” fruit peel were further validated in the KK-Ay diabetic mouse model. These metabolites not only effectively improved persistent hyperglycemia and glucose tolerance but also showed positive effects in lipid metabolism regulation and liver function protection.

Xiao et al. (2013) explored the hypoglycemic effects and potential mechanisms of citrus peel flavonoids in alloxan-induced diabetic mice. Treatment with citrus peel flavonoids significantly reduced fasting blood glucose levels in diabetic mice while increasing the activity of the antioxidant enzyme SOD, decreasing lipid peroxidation product MDA levels, and enhancing spleen and thymus function. These findings suggest that the hypoglycemic effects are closely related to the enhancement of antioxidant capacity, free radical scavenging, and immune system enhancement. Furthermore, diabetes is often accompanied by lipid metabolism disorders, including elevated serum TG, TC, and LDL-C levels, and decreased HDL-C levels. These metabolic disturbances are key contributors to severe cardiovascular complications such as coronary heart disease in diabetic patients. Citrus peel total flavonoids significantly reduced TC, TG, and LDL-C levels while increasing HDL-C levels, improving lipid metabolism disorders in diabetic mice. This further suggests that the hypoglycemic effects may be related to the regulation of lipid metabolism. By enhancing antioxidant capacity, scavenging free radicals, improving immune function, and ameliorating lipid metabolism disorders, citrus peel flavonoids not only effectively lower blood glucose but also show significant potential in the prevention and treatment of diabetes and its complications. However, the hypoglycemic effect of citrus flavonoids is relatively weak and cannot be used alone as the primary antidiabetic medication. They generally need to be combined with other antidiabetic drugs to achieve better blood sugar control.

### 5.2 Improvement of NAFLD

Non-alcoholic fatty liver disease (NAFLD) is a common chronic liver disease characterized by fat accumulation in the liver, but its onset is not due to excessive alcohol consumption. It is closely associated with metabolic syndrome, type 2 diabetes, obesity, and insulin resistance (Byrne and Targher, 2015; Pouwels et al., 2022). The disease can progress over time, increasing the risk of cirrhosis, end-stage liver disease, and hepatocellular carcinoma (Dufour et al., 2020). Globally, the prevalence of NAFLD is approximately 25% (Younossi et al., 2019). Naringin has shown significant protective effects in alleviating non-alcoholic fatty liver disease (NAFLD). The autophagic degradation of lipid droplets, known as lipophagy, is the primary mechanism of lipid consumption in hepatocytes. Guan et al. (2021) demonstrated that naringin significantly reduced hepatic fat accumulation and inflammation induced by a high-fat diet in mice by restoring autophagic flux and enhancing lipophagy. *In vitro* experiments also showed that naringin enhanced lipid breakdown by promoting transcription factor EB (TFEB)-mediated lysosome biogenesis and the fusion of autophagosomes with lysosomes, thereby reducing lipid accumulation. However, in TFEB knockout mice and hepatocyte models, the aforementioned effects of naringin were abolished, indicating the critical role of TFEB in naringin’s action against NAFLD. Zhang et al. (2021) showed that naringin significantly improved lipid metabolism in a NAFLD model. Naringin reduced intracellular triglyceride (TG) levels by 52.7% (from 0.36 ± 0.05 mM/g protein to 0.17 ± 0.03 mM/g protein, *P* < 0.05) and decreased very low-density lipoprotein (VLDL) secretion by 24.7% (from 10.24 ± 1.006 nmol/mL to 7.71 ± 0.576 nmol/mL, *P* < 0.05). Additionally, naringin downregulated key proteins associated with fatty acid uptake and *de novo* lipogenesis, such as cluster of differentiation 36 and acetyl-CoA carboxylase, and enhanced fatty acid β-oxidation by upregulating carnitine palmitoyltransferase 1 (CPT-1) and peroxisome proliferator-activated receptor alpha (PPAR-α). Molecular docking analysis revealed that naringin directly interacts with CD36 and PPAR-α, further supporting its role in reducing lipid accumulation and promoting fat oxidation. However, the TEF model may not fully reflect the dynamic changes in the human liver during long-term metabolic disorders. Citrus total flavonoids (PTFC) have been shown to alleviate liver inflammation and lipid deposition by regulating the TLR/CCL signaling pathway, providing a new perspective for the treatment of NAFLD (Wu et al., 2017).

### 5.3 Neuroprotective activity

Alzheimer’s disease (AD) is the most common age-related neurodegenerative disease, characterized by amyloid plaques, neurofibrillary tangles, and neurodegenerative lesions. Parkinson’s disease (PD) is the second most common, characterized by the loss of dopaminergic neurons in the substantia nigra. Nakajima and Ohizumi (2019) studied the effects of Nobiletin, a metabolite extracted from citrus peel, on Alzheimer’s and Parkinson’s diseases. They found that Nobiletin could improve neurodegenerative lesions through multiple mechanisms. The study indicated that Nobiletin reduces amyloid plaque accumulation in AD models by inhibiting β-secretase activity and promoting amyloid protein degradation, thereby alleviating its toxic effects on neurons. It also promotes synaptic plasticity and neuronal function recovery by regulating the cAMP/PKA/ERK/CREB signaling pathway. Additionally, its antioxidant, anti-inflammatory, and endoplasmic reticulum stress-relieving effects significantly reduced oxidative stress and neuroinflammation levels in both AD and PD models, protecting neurons from inflammatory damage. In PD models, Nobiletin restored dopamine signaling, reduced neuronal loss, and improved motor dysfunction.

The incidence of Parkinson’s disease is steadily increasing, but its etiology and pathogenesis are not yet fully understood. However, excessive neuronal apoptosis is thought to be closely related to the disease. Naringin and naringenin have shown significant neuroprotective potential in the treatment of Parkinson’s disease. He et al. (2021) demonstrated that naringin and naringenin regulate the expression of long-chain non-coding RNA SNHG1 and further modulate its downstream molecular mechanisms. For example, downregulation of small nucleolar RNA host gene (SNHG) can reduce its binding to specific microRNAs (such as miR-7 or miR-124), thereby releasing the activity of these microRNAs and inhibiting the expression of pro-inflammatory factors and apoptosis-related genes. This regulatory effect not only alleviates neuroinflammation and mitochondrial damage but also promotes neuronal survival and inhibits apoptosis by modulating the PI3K/AKT signaling pathway. However, research on other potentially involved signaling pathways, such as NF-κB or Nrf2-ARE, is still insufficient. Kim et al. (2016) used 8-week-old C57BL/6 mice to establish a Parkinson’s disease neurotoxic model by unilaterally injecting 6-hydroxydopamine (6-OHDA) into the striatum. They investigated whether naringin had neuroprotective and neurorestorative effects on the nigrostriatal dopamine projection. The experiment included both pre-treatment and post-treatment approaches, where naringin was administered before the 6-OHDA injection and 3 weeks after. The study found that naringin significantly alleviated the neurotoxicity induced by 6-OHDA, including reducing neuronal death and fiber loss in the nigrostriatal dopamine projection. Moreover, naringin exhibited both anti-inflammatory and neuroprotective effects by activating mammalian target of rapamycin complex 1 (MTORC1) and inhibiting microglial activation. However, current research has many limitations, and further experimental validation and clinical studies are needed to fully assess its translational medical value.

### 5.4 Antioxidant, anti-inflammatory and antimicrobial effects

#### 5.4.1 Antioxidant

Naringin and hesperidin are the primary flavonoids in the citrus family, and they can exert effective antioxidant effects by being converted into naringenin and hesperetin, which then influence various signaling pathways in the body (Razavi and Hosseinzadeh, 2019). Madeira and Macedo (2015) used hesperidin and naringin as raw materials and employed enzymatic extraction and biotransformation (cellulase, pectinase, and tannase) to obtain hesperetin and naringenin. The study found that their antioxidant activity *in vitro* was significantly stronger than that of their glycoside forms (Xie et al., 2022). Guo et al. (2020) confirmed through several *in vitro* studies using antioxidant evaluation methods that citrus flavonoids extracted with ethyl acetate (EtOAcE) are effective natural antioxidants. Ferlazzo et al. (2016) investigated the antioxidant effects of citrus extracts rich in flavonoids in an Fe2(SO4)3-induced A549 cell model. The study showed that citrus extracts exhibited significant antioxidant activity. The levels of ROS induced by ferric sulfate increased 6–13 times, and lipid peroxides and DNA damage were significantly elevated, while mitochondrial function was impaired. However, citrus extracts significantly reduced ROS production (by 50%–70%), lipid peroxides (by 55%–73%), and DNA damage (by 65%–70%). The mechanism of action may be related to the chelating properties of iron and the increased antioxidant enzyme activity, including catalase, in the flavonoid extracts.

#### 5.4.2 Anti-inflammatory and antimicrobial

Naringin is a potential antibiofilm agent that can be used in combination with antibiotics to develop drugs for the treatment of biofilm-related infections. Naringin has been shown to enhance the antibacterial effects of ciprofloxacin and tetracycline against *Pseudomonas aeruginosa* biofilms. Dey et al. (2020) demonstrated that Naringin exhibited significant biofilm clearance effects at sub-minimum inhibitory concentrations (sub-MIC), with the combination of naringin and ciprofloxacin achieving up to 89.35% biofilm clearance, as shown in crystal violet and MTT assays. Furthermore, Congo red binding assays and microscopic imaging confirmed that the naringin combination significantly reduced the extracellular polymeric substance (EPS) matrix and thickness of the biofilm. Compared to antibiotics alone, the combination therapy showed better biofilm clearance on indwelling catheter surfaces and exhibited lower toxicity to mouse macrophages, with cell viability exceeding 95%.

Naringin can significantly counteract the detrimental effects of TNF-α on NP cells and demonstrate strong anti-inflammatory activity in an *in vitro* intervertebral disc degeneration (IDD) model. Chen et al. (2022) found that naringin could significantly reduce the TNF-α-induced inflammation in human NP cells. After 24 h of TNF-α treatment, the level of COX-2 in NP cells was significantly elevated, while in cells pretreated with naringin, COX-2 levels were effectively suppressed (Wang et al., 2021). In an acidic environment, which is typical of localized inflammation, TNF-α-treated cells exhibited lower pH compared to untreated or DMSO-treated control groups, while naringin pretreatment significantly increased the pH. He et al. (2018) found that naringenin, nobiletin, and hesperetin exert anti-inflammatory effects on LPS-induced RAW264.7 cells, and the mechanism may be related to the TNF-α-induced NF-κB pathway, as well as COX-2 and iNOS regulation.

### 5.5 Cardiovascular protective effects

In recent decades, cardiovascular diseases have been one of the leading causes of death worldwide. In the past 2 years, it has been estimated that approximately 330 million people globally suffer from cardiovascular diseases, and there is still a large population in China with cardiovascular risk factors. Both the incidence and mortality rates of cardiovascular diseases are continuously rising (Wang et al., 2023). Naringin and naringenin have potential roles in the prevention and treatment of cardiovascular diseases. Both are flavonoid metabolites widely found in citrus fruits, especially grapefruits. Heidary Moghaddam et al. (2020) demonstrated that in a high-cholesterol diet rabbit model, naringin and naringenin could downregulate the gene expression of monocyte chemoattractant protein-1 (MCP-1) and vascular cell adhesion molecule-1 (VCAM-1), reducing atherosclerotic plaque formation. In a high-glucose environment, naringin effectively reduced cardiomyocyte apoptosis by inhibiting oxidative stress and the mitogen-activated protein kinase (MAPK) signaling pathway. Additionally, naringenin alleviated cardiac hypertrophy by activating the AMPK-mTOR signaling pathway and improved endothelial function. In rat experiments, naringenin also showed protective effects against myocardial ischemia-reperfusion injury by regulating mitochondrial-related signaling pathways to improve heart function. Naringin improved myocardial ischemia-reperfusion injury through the miR-126/GSK-3β signaling pathway. Guo et al. (2022) demonstrated that in a rat myocardial ischemia/reperfusion (I/R) model, treatment with naringin or miR-126 agomir combined significantly alleviated cardiac inflammation, reduced cardiomyocyte apoptosis, and notably suppressed the expression of inflammatory factors (IL-6, IL-8, and TNF-α). Moreover, naringin or miR-126 agomir regulated the GSK-3β signaling pathway by lowering the phosphorylation levels of GSK-3β, promoting β-catenin entry into the nucleus, thereby exerting a protective effect on the myocardium.

Atherosclerosis (AS) is a chronic inflammatory cardiovascular disease characterized by lipid deposition in the vessel wall and immune cell recruitment, and it is a major risk factor for cardiovascular diseases (Liu et al., 2023). Yu et al. (2022) found that in mice fed a high-fat diet, oral administration of naringin significantly reduced total cholesterol and triglyceride levels while increasing high-density lipoprotein cholesterol levels. Li et al. (2014) showed that naringin upregulated the expression of superoxide dismutase (SOD), downregulated malondialdehyde (MDA) levels, and protected endothelial cells from TNF-α-induced damage by blocking oxidative stress, thereby improving the development of atherosclerosis.

### 5.6 Regulation of lipid metabolism

Lipid metabolism refers to the series of biochemical reactions involved in the digestion, absorption, transport, storage, and breakdown of lipid substances in the body, such as triglycerides, cholesterol, phospholipids, and free fatty acids. Imbalance in lipid metabolism can lead to diseases such as obesity, fatty liver, hyperlipidemia, and atherosclerosis.

The digestion and absorption of dietary fats primarily occur in the small intestine, and this process depends on the activity of pancreatic lipase (PL) in the duodenum and jejunum. Pancreatic lipase is the key enzyme responsible for triglyceride hydrolysis in the gastrointestinal tract. Therefore, inhibiting PL activity is considered a potential strategy to reduce fat absorption and restore lipid homeostasis. Among them, natural product lipase inhibitors such as naringin and hesperidin have gradually become a research focus due to their safety and diversity (Liu et al., 2020; Rajan et al., 2020). Huang et al. (2020) analyzed eight citrus peel extracts and found that hesperidin exhibited the most significant pancreatic lipase inhibition and antioxidant activity. It interacts with lipase through hydrogen bonds and van der Waals forces, making it a potential natural ingredient for free radical scavenging and controlling obesity. Rotimi et al. (2018) used a high-fat, low-chain uric acid rat T2DM model and found that naringin showed significant anti-diabetic effects and improved lipid metabolism in the diabetes model. Naringin dose-dependently reduced plasma glucose, DPP-IV levels, and renal ACE activity, while significantly increasing paraoxonase (PON) activity, particularly in plasma and liver. Naringin also lowered cholesterol levels, inhibited the increase in PON activity in VLDL and VLDL 3, and reversed the decline in HDL3 cholesterol. Additionally, it restored the decrease in Carnitine Palmitoyltransferase 1(CPT -1) activity caused by diabetes and regulated the gene expression of liver genes such as Scarb1 and Ahr.

### 5.7 Gut protective effects

Inflammatory bowel disease (IBD) is an uncontrollable, non-specific chronic immune-mediated intestinal inflammation, which can be divided into Crohn’s disease (CD) and ulcerative colitis (UC) (Kaplan, 2015). The pathogenesis of UC is not yet fully understood. The general consensus is that UC is closely associated with immune system dysfunction, intestinal mucosal damage, and dysbiosis in genetically susceptible populations. Naringin has shown significant therapeutic effects on UC. Cao et al. (2021) used a dextran sulfate sodium (DSS)-induced mouse model of ulcerative colitis and found that naringin reduced the Disease Activity Index (DAI) score, alleviated colon tissue damage, and exerted effects through oxidative stress and inflammatory factors. Additionally, naringin increased the expression of tight junction proteins in the mucosa, improved the relative abundance of *Firmicutes/Bacteroidetes*, and reduced the levels of Proteobacteria, thus improving gut microbiota dysbiosis. The integrity of the intestinal barrier is crucial for defending against pathogen invasion. When the body is exposed to various environmental factors, the intestinal barrier may be compromised, allowing the entry of foreign pathogens and endotoxins, thereby jeopardizing health Zhang et al. (2022). Naringin can alleviate lipopolysaccharide (LPS)-induced intestinal barrier damage in mice. Luo et al. (2023) demonstrated that naringin effectively reduced LPS-induced intestinal barrier damage by inhibiting inflammatory factors, improving antioxidant function, and enhancing the expression of intestinal tight junction proteins. This protective effect may be mediated by the activation of the Nrf2 signaling pathway and inhibition of the TLR4/p38 MAPK/NF-κB signaling pathway. Experimental results showed that LPS significantly increased serum diamine oxidase (DAO) activity, D-lactic acid (D-LA) concentration, and malondialdehyde (MDA) levels, while reducing the activity of antioxidant enzymes (SOD, Gpx, CAT) and causing damage to intestinal tissue morphology and tight junction protein expression (ZO-1, Occludin, Claudin). LPS also upregulated the expression of inflammatory factors (TNF-α, IL-1β, IL-6) and genes related to TLR4/p38 MAPK/NF-κB signaling. However, naringin significantly reversed these changes, thereby protecting the intestinal barrier (Carroll et al., 2003). Nevertheless hesperidin, as a natural product, has a low bioavailability, meaning its absorption, distribution, metabolism, and excretion in the body may be limited. This can result in a decreased therapeutic efficacy in clinical treatment.

## 6 Conclusion

Citrus flavonoids, as natural phytochemicals, have demonstrated a wide range of health benefits, particularly in the areas of antioxidant, anti-inflammatory, anti-diabetic, lipid-lowering, and protection of the gut and cardiovascular system. In recent years, significant progress has been made in the study of the biological activities and molecular mechanisms of citrus flavonoids, providing new strategies for the prevention and treatment of metabolic diseases such as diabetes and NAFLD. At the same time, advancements in extraction and purification technologies have facilitated the development and application of citrus flavonoids in the fields of food, medicine, and functional materials. Future research should focus on further elucidating the mechanisms of action of citrus flavonoids and their safety and efficacy in clinical applications, ultimately contributing to greater benefits for human health.
